# Oral health, antimicrobial resistance and the need for interprofessional education

**DOI:** 10.3389/froh.2026.1894083

**Published:** 2026-07-07

**Authors:** Jelena Roganović, Ivana Šutej, Milena Barać, Marijana Radić Vuleta, Dragica Manojlović, Danilo Pavlović, Vojislav Komlenić, Jugoslav Ilić, Katarina Radović

**Affiliations:** 1 Department of Pharmacology in Dentistry, School of Dental Medicine, University of Belgrade, Belgrade, Serbia; 2 Department of Pharmacology, School of Dental Medicine, University of Zagreb, Zagreb, Croatia; 3 Croatian Institute of Public Health, Zagreb, Croatia; 4 Department of Restorative Odontology and Endodontics, School of Dental Medicine, University of Belgrade, Belgrade, Serbia; 5 Department of Prosthetic Dentistry, School of Dental Medicine, University of Belgrade, Belgrade, Serbia

**Keywords:** antibiotic stewardship, antimicrobial resistance, dental prescribing, interprofessional education, oral health

## Abstract

This mini review examines how oral microbial ecology, dental antibiotic prescribing, fragmented clinical pathways at the medical–dental–pharmacy interface, and ethical pressures together contribute to antimicrobial resistance (AMR) in oral healthcare, and argues that interprofessional collaboration and interprofessional education are essential components of an effective response. Relevant literature was identified through targeted searches of PubMed/MEDLINE, Scopus, Web of Science, Google Scholar, and the Cochrane Library**,** relevant guidelines and policy documents resulting in selection of seventy-two references comprising four thematic domains: clinical coordination and decision-making across dental, medical, and pharmacy settings; antimicrobial stewardship and rational antibiotic use in dentistry; fragmented care pathways at the medical–dental–pharmacy interface; and ethical and educational dimensions of prescribing. The novelty of this review lies in bringing these domains together within a single conceptual framework that positions oral healthcare not as a peripheral issue, but as biologically, clinically, and ethically important site of AMR emergence and stewardship.

## 1 Introduction

Antimicrobial resistance (AMR) is one of the most urgent global threats to health, healthcare systems, and sustainable development. In 2021, bacterial AMR was associated with approximately 4.71 million deaths worldwide, including 1.14 million deaths directly attributable to resistant bacterial infections (1). Inappropriate, excessive, poorly targeted, or poorly coordinated antibiotic use creates selective pressure that supports the survival and spread of resistant bacteria through mutation, vertical transmission, and horizontal gene transfer (2, 3). Although AMR is commonly framed through medicine, microbiology, hospital stewardship, and systemic infectious disease, the oral cavity is also highly relevant because it contains one of the most complex microbial communities in the human body and may serve as a reservoir for microorganisms capable of exchanging resistance genes (2, 3). Dentistry contributes to AMR not only through prescribing frequency, but also through the biological and clinical features of oral disease. Dental prescribing accounts for approximately 10% of all antibiotic prescriptions, and a substantial proportion of these prescriptions, reported in some studies as up to 90%, may be unnecessary, inappropriate in indication, too broad in spectrum, or longer than needed (4, 5). Oral infections often develop within dense biofilms and may be chronic or recurrent, creating conditions in which resistant organisms and resistance genes can persist (2, 3). Self-medication, incomplete adherence, patient expectations that antibiotics should be used for dental pain or swelling, and dental procedures involving close contact with saliva, blood, instruments, shared surfaces, and aerosols may further contribute to inappropriate antibiotic use and potential transmission risks (6). Because oral and systemic health are interconnected, antibiotic decisions in dentistry may involve factors beyond the oral condition itself, including comorbidities, polypharmacy, allergy history, previous antibiotic exposure, drug interactions, and risk of systemic complications. In fragmented care pathways, patients with oral infections may move between dental, medical, pharmacy, specialist, and hospital settings, while relevant information remains disconnected. Physicians, pharmacists, dentists, and, in the wider One Health context, veterinary professionals, all contribute to antibiotic exposure, patient expectations, prescribing culture, and the broader ecology of resistance (7). Therefore, antimicrobial stewardship in oral healthcare should not be understood as a profession-specific responsibility alone, but as a shared clinical, educational, and public health task requiring interprofessional collaboration (7). In this manuscript, interprofessional collaboration and interprofessional education (IPE) are related but distinct concepts. Interprofessional collaboration refers to how dentists, physicians, pharmacists, and other professionals coordinate care in practice, for example through referral, medication review, allergy clarification, and shared stewardship protocols. IPE refers to how students or clinicians learn with, from, and about each other in order to build role clarity, communication skills, ethical reasoning, and readiness for collaborative practice. The review therefore treats collaboration as a clinical and organizational response, and IPE as an educational mechanism that may prepare professionals for that response.

Nevertheless, previous literature has generally focused on one segment of the complexity of rational antibiotic prescribing in oral healthcare at a time, such as dental antibiotic prescribing, stewardship interventions, oral biofilm and resistome biology, or interprofessional education and collaboration. Consequently, these areas have largely developed as parallel bodies of literature rather than as an integrated framework explaining how biological, clinical, behavioral, and professional factors interact in oral healthcare stewardship. The novelty of the present review lies in integrating these parallel perspectives into a shared explanatory framework. This approach helps identify where stewardship failures overlap such as: biological complexity in oral biofilms, delayed or inadequate source control, fragmented medical–dental–pharmacy pathways, patient pressure for antibiotics, and unclear professional responsibility across care settings.

## 2 Method

This article was conducted as a narrative review examining AMR in oral healthcare, with particular focus on dentistry, oral microbial ecology, antibiotic prescribing, fragmented medical–dental–pharmacy pathways, ethical challenges, and the role of interprofessional collaboration and interprofessional education (IPE). Relevant literature was identified through targeted searches of PubMed/MEDLINE, Scopus, Web of Science, Google Scholar, and the Cochrane Library, supplemented by screening of reference lists and consultation of relevant guidelines and policy documents from organizations such as the American Dental Association, FDI World Dental Federation, and WHO. Search terms included: antimicrobial resistance, oral microbiome, oral biofilm, oral resistome**,** dentistry antibiotic prescribing**,** dental antibiotic stewardship**,** odontogenic infection**,** infective endocarditis prophylaxis**,** general practitioners and dental pain**,** community pharmacists and oral health, interprofessional collaboration**,** interprofessional education**,** and ethics of antibiotic prescribing. The articles were included if they addressed one or more of the following: AMR in relation to oral health or oral ecology; antibiotic prescribing in dentistry; stewardship interventions or prescribing behavior; management of dental conditions in medical settings; pharmacists’ contribution to oral health and antibiotic stewardship; ethical issues in prescribing; or interprofessional collaboration and IPE relevant to stewardship. A total of 157 records and documents were initially identified. After removal of 28 duplicates, 129 records were screened by title, abstract, document type, and apparent relevance. Forty-one records were excluded because they were outside the scope. Eighty-eight full texts or policy/guideline documents were assessed for eligibility. Sixteen were excluded because of insufficient overlap with stronger or more recent sources, narrow microbiological focus without clinical or educational relevance, or limited applicability to AMR stewardship in oral healthcare. Finally, seventy-two references were included, and synthesized thematically into domains reflecting the key dimensions of antimicrobial resistance in oral healthcare. These comprised four domains that underpin the need for interprofessional collaboration and education as a cross-cutting response to antimicrobial resistance in oral healthcare: (1) clinical coordination and decision-making across dental, medical, and pharmacy settings; (2) antimicrobial stewardship and rational antibiotic use in dentistry; (3) fragmented care pathways at the medical–dental–pharmacy interface; (4) ethical and educational dimensions of prescribing (Table 1).

**Table 1:** Main domains and key references supporting the role of interprofessional collaboration in antimicrobial stewardship in oral healthcare.

Domain	Why interprofessional collaboration matters	Key references from the text
1. Clinical coordination and better decision-making	Patients with oral infections often visit dental, medical, and pharmacy settings. Collaboration improves assessment of indication for antibiotics, systemic risk, comorbidities, allergy status, drug interactions, and the need for referral or definitive treatment.	Caivano et al. (7); Lockhart et al. (20); Segura-Egea et al. (21); Teughels et al. (22); Wilson et al. (23); Suda et al. (24); Stojilković et al. (69); Juárez-Membreño et al. (47); de Oliveira et al. (48)
2. Antimicrobial stewardship and rational antibiotic use	Collaboration strengthens stewardship by reducing unnecessary prescribing, improving guideline adherence, supporting narrow-spectrum and better-targeted therapy, and preventing antibiotics from being used instead of causal, e.g., endodontic or surgical treatment	Soleymani et al. (4); Sukumar et al. (33); Teoh et al. (16); Tolksdorf et al. (30); Mendez-Romero et al. (31); Thabit et al. (29); Lockhart et al. (20); Segura-Egea et al. (21); Šimundić Munitić et al. (25); Šutej et al. (26); Šutej et al. (27); Sović et al. (28); D’Ambrosio et al. (32); Okihata et al. (56); Schmid et al. (57)
3. Overcoming fragmented care pathways	Interprofessional collaboration is needed because patients with dental pain or swelling often enter fragmented pathways, including general practice, emergency care, or pharmacies, while definitive dental treatment may be delayed and antibiotics may be used unnecessary	Teoh et al. (34); Biezen et al. (35); Rajiah et al. (36); Jones et al. (37); Lygre et al. (38); Thabit et al. (29); El Said et al. (39); Holzinger et al. (58)
4. Ethical and educational responsibility	Collaboration has ethical significance because inappropriate prescribing is influenced not only by knowledge gaps, but also by professional boundaries, patient expectations, and uncertainty in level of responsibility. Interprofessional education helps build ethical reasoning, role clarity, and a shared stewardship understanding and implementation.	Roganović and Barać (11); Roganović (46); Thompson et al. (14); Cope et al. (40); Newlands et al. (15); Teoh et al. (42); Dormoy et al. (43); Vázquez-Cancela et al. (41); General Dental Council (44); Hughes et al. (45); Yen and Cutrell (70); Adebisi (71); Jamrozik and Heriot (72); Reeves et al. (18); Barr and Low (49); Oudbier et al. (50); Huebner et al. (51); MacDougall et al. (53); Guilding et al. (54); El Said et al. (39)

## 3 Oral health and relevance for antimicrobial resistance

Oral health represents functional, structural, and psychosocial state in which the oral cavity is free from pain, infection, and significant disease. In context of anatomical environments, the oral cavity contains multiple distinct ecological niches, including the tongue, saliva, supragingival and subgingival plaque, mucosal surfaces, and periodontal pockets. Each niche supports different microbial communities and enables intense microbial interaction. This ecological diversity facilitates the exchange of mobile genetic elements and makes the oral cavity a potentially important reservoir of the antimicrobial resistance genes present in oral microorganisms- resistome (2, 3). Oral microbiome contributes to homeostasis, but if disrupted, dysbiosis promotes the development of oral diseases including caries, gingivitis, periodontitis, peri-implantitis, and denture-related infections (8, 9). The central challenge for dentistry is therefore not merely to suppress infection, but to preserve or restore oral health while minimizing selective pressures that could foster AMR (8, 9).

Noteworthy, oral microorganisms organize into structured biofilms embedded in an extracellular matrix on teeth, mucosa, restorations, dentures, implants, and even dental unit waterlines (8, 10). These biofilms are more tolerant to host defenses and to antimicrobials, partly because the matrix limits drug penetration and partly because biofilm-associated organisms may have reduced metabolic activity and enhanced opportunities for horizontal gene transfer (8, 10). As a result, the oral environment becomes a favorable setting not only for persistent infection, but also for the maintenance, exchange, and spread of antimicrobial resistance genes (9, 10). In this sense, oral health management and AMR stewardship are inseparable: the better biofilms are prevented and mechanically controlled, the lower the need for systemic antibiotics and the lower the opportunity for resistance selection (8).

Although odontogenic infections can be controlled and managed through local operative measures such as abscess drainage and endodontic therapy, frequently antibiotics were used to compensate for clinical uncertainty, delayed care, or workflow pressures (8–15). The disturbed oral ecosystems and poorly governed dental antibiotic use may contribute not only to treatment failure, but also to broader circulation of resistance factors (8, 9).

Improving oral healthcare in the context of AMR requires accurate diagnosis, recognition of when local treatment is sufficient, and wider use of strategies that reduce antibiotic burden while preserving ecological balance. In this context, the continuous education for students, dentists, and patients on rational antibiotic use, resistance risks, and the oral-specific nature of AMR is essential part of oral healthcare (8, 9). Oral infections frequently occur in patients whose care extends beyond dentistry, especially those with chronic systemic diseases, polypharmacy, xerostomia, immunomodulation, or other medication-related effects that may disturb oral homeostasis. Such patients may seek care from dentists, physicians, and pharmacists at different points in the same clinical pathway. Therefore, the interprofessional collaboration is needed, while IPE may strengthen role clarity, prescribing confidence, medication review, and shared stewardship protocols (12, 16–18) to understand that AMR in dentistry is not a narrow dental issue, but part of a wider public health challenge (12, 13, 19).

## 4 Knowledge gaps and fragmented clinical pathways at the medical–dental–pharmacy interface

### 4.1 Dentists: stewardship knowledge gaps

In dentistry, systemic antibiotics are indicated mainly as an adjunct to local operative treatment, when odontogenic infection spreads beyond the local site, such as cellulitis, fascial space involvement, trismus, fever, or other systemic signs. Namely, ADA and ESE guidance emphasizes source control through endodontic treatment, incision, and drainage, while antibiotics should be reserved for systemic involvement or cases where drainage cannot be achieved (20, 21). In periodontology, systemic antibiotics may be appropriate only in selected severe cases, particularly stage III/IV or grade C periodontitis, as an adjunct to subgingival debridement (22). Prophylaxis is even more restricted and mainly applies to patients at highest risk of infective endocarditis undergoing invasive dental procedures (23, 24).

However, data shows that antibiotics, and often of a broader-spectrum, are used for conditions that usually require operative care alone, such as pulpitis, infected canals without periapical lesions, localized abscess with drainage, or uncomplicated extractions (20, 21, 25–27). A persistent gap also remains between guideline awareness and guideline-concordant practice. Many dental prescriptions are unnecessary or disproportionate, while dental stewardship still requires clearer guidance, audit, feedback, and continuing education (28–31).

Another important issue is underestimation of resistance risk. In one survey, most Italian dentists were aware of AMR, but fewer consistently consulted guidelines, and fewer than half believed their prescribing could influence resistance development (32). Since resistance is underlied by cumulative antibiotic exposure across healthcare, dental prescribing should not be treated as a small, isolated domain (4, 29, 33). Recent evidence therefore supports not only improved knowledge, but also system-level interventions such as audit and feedback, digital decision support, and structured collaboration with pharmacists (29, 31).

### 4.2 Physicians: when dental problems become medicalized

Fragmentation becomes clear when patients with dental problems enter medical instead of dental office. Dental pain is common in general practice, often because patients cannot access timely dental care. In this context, dental disease becomes medicalized: patients enter a system designed for symptom relief and prescribing, although the underlying problem usually requires dental treatment (34, 35). This helps explain why antibiotics are prescribed even when unlikely to help. Teoh et al. found that only 23.6% of antibiotics prescribed by general practitioners for dental pain, were guideline-concordant, although most dental pain and infection are resolved by dental treatment only (34). In that way, the frequent prescribing of antibiotics for pulpitis in both dental and medical settings represents a clear example of stewardship failure across different care modalities (32, 34, 35).General practitioners often know that antibiotics will not resolve dental problems, but they face limited oral health training, patient anxiety, cost barriers, long waiting times, and sometimes requests from dentists for pre-treatment antibiotics (35).

### 4.3 Pharmacists: the first point of contact

Pharmacists often encounter oral health problems early, since community pharmacies are accessible, familiar, and frequently used as informal first-contact settings, especially when dental care is delayed or difficult to access (36, 37). This makes pharmacists an important but underused stewardship interface. They commonly see oral or facial pain, swelling, ulcers, dry mouth, thrush, and denture problems; in one survey, oral or facial pain and swelling were encountered weekly by most respondents, and nearly one third saw oral or dental pain daily (37).

However, the role of pharmacists in dental stewardship is limited by insufficient training, uncertainty about referral thresholds, and weak integration with dental care. Rajiah et al. found that 44% of community pharmacists lacked sufficient knowledge to provide appropriate recommendations for dental problems, although most were willing to improve their knowledge. Jones et al. similarly reported difficulties with examination, diagnosis and referral pathways (36, 37). Some pharmacists do not routinely refer patients with oral health problems to dentists, and do not consult dentists (36).

Pharmacists could help identify when antibiotics are unlikely to solve the problem, discourage self-medication intentions, and refer patients urgently to dentist when swelling, spreading infection, or other symptoms are present. This role requires adequate training, shared protocols, and clear referral thresholds (29, 37). Interprofessional collaboration and IPE involving dentistry and pharmacy can improve communication, role understanding, and collaborative competencies relevant to antimicrobial management and referral behavior (38, 39).

## 5 Ethical and educational challenges in antibiotic prescribing in oral healthcare

Antibiotic prescribing in dentistry raises ethical concerns beyond indication and dosage, because it affects patient safety, professional integrity, and AMR control. When prescribing is shaped by patient pressure, time constraints, fear of complaints, or avoidance of conflict, antibiotics may become a substitute for communication, operative care, or professional confidence rather than a clinically justified intervention (11, 14, 15, 40). This undermines non-maleficence, weakens accountability, and contributes to inappropriate antibiotic use in population.

Moreover, prescribing under patient expectation or professional anxiety may normalize antibiotics as a convenient response to dissatisfaction, delay, or uncertainty (41–43). Similarly, prescribing for non-dental conditions, doctor's relatives, or under pressure from other professionals, blurs professional boundaries, objectivity, and responsibility (11, 44, 45). IPE may be relevant for dental antibiotic stewardship via several ethical issues: professional responsibility, non-maleficence, patient autonomy, justice, role clarity, and accountability. Namely, learning with medical, pharmacy, and other healthcare students can help dental students distinguish legitimate collaboration from inappropriate transfer of responsibility. It can also teach them how to refuse unnecessary antibiotics while preserving patient trust, how to explain why source control is preferable to medication alone, how to recognize when referral or consultation is necessary, and how to manage disagreement across professional boundaries. In this sense, IPE supports ethical reasoning by linking antimicrobial stewardship to communication, role clarification, professional courage, shared accountability, and protection of antibiotics as a public-health resource. Fostering IPE among students and trainees is especially beneficial for AMR, as habits formed early persist well in the clinical practice (11, 46).

## Discussion

The four domains identified in this review should not be interpreted as separate themes, but as interacting levels of the same stewardship problem. Oral microbial ecology explains why prevention, biofilm control, and source control matter biologically and systematically: persistent oral biofilms and resistome reservoirs create conditions in which unnecessary antibiotic exposure can select for resistant oral microorganisms or resistance genes which contribute to broader microbial dysbiosis, complicate the management of medically vulnerable patients, and increase the risk of difficult-to-treat infections when oral bacteria disseminate or interact with systemic disease (2, 3, 8–10). Dental prescribing guidance establishes the clinical basis of stewardship in oral healthcare (20, 21), but without source control, antibiotic use may be both clinically insufficient and microbiologically harmful, particularly in oral biofilms. These clinical and microbiological challenges become more difficult to manage when care pathways are fragmented. The guideline-concordant decisions may fail in practice because patients with dental pain or infection often seek help from GPs, emergency departments, or pharmacies when access to definitive dental care is delayed. In these settings, clinicians may have only partial information, limited knowledge of the dental source of infection, and limited capacity to provide drainage or other source-control procedures (34–38). The ethical and educational domain explains why this pattern persists even when professional knowledge exists: patient pressure, diagnostic uncertainty, professional anxiety, poor role clarity, and transferred responsibility may push clinicians toward antibiotics as the easier, faster, or more defensible response (11, 14, 15, 40–45).The overlap between these domains is therefore the central point of the framework. Stewardship failure occurs most clearly at the interfaces: when pulpal or periapical disease is medically managed without dental source control; when pharmacists encounter oral symptoms but lack referral pathways; when dental guidelines do not fully address comorbidity, allergy history, polypharmacy, or drug-drug interactions (29, 34–37, 47, 48). Interprofessional collaboration addresses the practice-level interface by improving referral, medication review, allergy clarification, and consistent patient messaging. IPE addresses the training-level interface by preparing future professionals to understand each other's roles, communicate across boundaries, and treat antibiotics as a shared public-health resource rather than as a profession-specific convenience (18, 49–54).

Available intervention evidence supports this distinction. Multidisciplinary care pathways, pharmacist involvement in antimicrobial-stewardship programs, audit and feedback, may support prescribing behaviour (29, 31, 55–57). While collaboration changes how care is delivered now, IPE prepares professionals to collaborate more appropriately in future practice.

IPE curricula and interprofessional workshops primarily aim to improve knowledge, attitudes, communication skills, role understanding, and readiness for collaborative practice (39, 50–54). In the context of AMR, this makes IPE relevant to oral healthcare because stewardship-oriented learning can prepare dental, medical, pharmacy, and other healthcare students to coordinate prescribing decisions, clarify professional responsibilities, and support rational antibiotic use across care pathways (39, 58–62). Although evidence on medicine-dentistry IPE remains limited, existing studies suggest that structured interprofessional activities can improve students' attitudes, teamwork understanding, collaborative readiness, antibiotic-stewardship knowledge, and recognition of oral healthcare's role in antimicrobial stewardship (63–72).

This review has several limitations. First, as a narrative review, it did not involve participant-level data collection; therefore, sex- or gender-disaggregated analysis could not be performed. Second, much of the available literature comes from Europe, Australia, North America, and other high-resource settings, which may limit the transferability of the proposed framework to other contexts. The framework should therefore be tested, refined, and adapted in low- and middle-income countries, where the AMR burden, over-the-counter antibiotic access, delayed access to dental care, workforce shortages, and stewardship infrastructure may differ substantially.

## Conclusions

**Figure 1:**
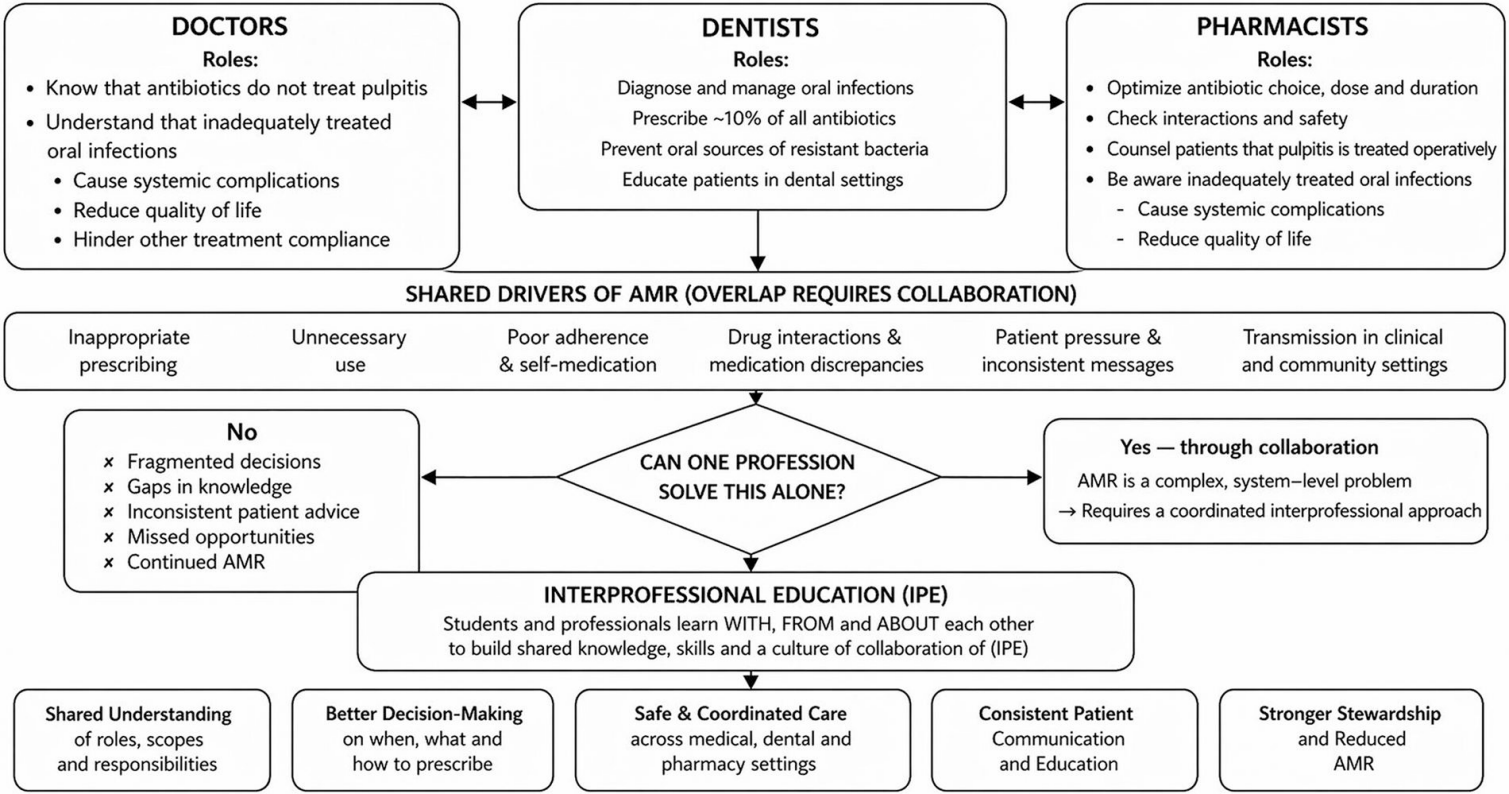
Conceptual framework illustrating the role of interprofessional collaboration in addressing antimicrobial resistance in oral healthcare.

The present review supports two related but distinct responses to AMR in oral healthcare: interprofessional collaboration in clinical practice and IPE by strengthening role clarity, guideline-concordant prescribing, source-control reasoning, patient communication, accountability, and shared responsibility in complex oral healthcare pathways (Figure 1). In practical terms, interprofessional collaboration should not be understood as requiring dentists to consult another professional before every antibiotic prescription. Rational antibiotic prescribing remains a core dental responsibility, and dentists must know and apply current indications and guidelines. However, collaboration is most relevant when prescribing decisions associate with systemic disease, polypharmacy, unclear allergy history, medication interactions, high-risk cardiac conditions, delayed access to definitive dental treatment, or hospital-based care.
